# Niche overlap between 2 small sympatric predators from the Atacama Desert

**DOI:** 10.1093/cz/zoag007

**Published:** 2026-02-12

**Authors:** Andrés Taucare-Ríos, Jheremy Méndez-Yovera

**Affiliations:** Facultad de Ciencias, Universidad Arturo Prat, Casilla 121, Iquique 1100000, Chile; Facultad de Ciencias, Universidad Austral de Chile, Av. Rector Eduardo Morales Miranda 23, Valdivia 5090000, Región de Los Ríos, Chile

**Keywords:** arid environments, Chile, competitors, microhabitat

The niche of a species may be characterized as a measure of resource utilization (Schoener 1974). Niche overlap describes the situation in which co-occurring species share parts of their niche space with each other. High niche overlap may lead to competition and exclusion for some species although low niche overlap implies niche segregation or lower levels of interaction, allowing for sustainable species coexistence (Schoener 1974; Yang and Hui 2021). In this sense, some authors have observed that in experimental studies, overlapping in different niche dimensions tends to be positively associated with strong competition (Schoener 1983).

Direct interactions between sympatric competitors, such as interference competition, can lead to spatial and temporal avoidance (Polis et al. 1989). Under the competitive exclusion principle, species should partition niche dimensions (Pianka 1973; Schoener 1974) that enables stable coexistence (Case and Gilpin 1974). If the competitor species overlap on one dimension, then they should differ on another niche dimension (Schoener 1974). Spatial and temporal niche segregation likely arise as adaptations to reduce both exploitation and interference competition between species (Carothers and Jaksić 1984; Pastore et al. 2021; Kachel et al. 2022). Species coexistence is mainly influenced by mechanisms acting on niche differences, highlighting the importance of niche segregation (Schoener 1974; Pastore et al. 2021; Buche et al. 2022).

Body size often influences competitive ability and resource use; therefore, large individuals (dominant) usually are better competitors than smaller species (subordinate) (Young 2004). In arid environments, where prey availability is restricted, a high degree of overlap may suggest intense exploitation competition among predators of different body sizes (Young 2004; Kachel et al. 2022).

Temperature is particularly important for ectotherms assessing the suitability of foraging and reproduction habitats, especially where females remain long time within their shelters (Angilletta et al. 1999; Pérez and Balta 2011; Vidal et al. 2011; Taucare-Ríos et al. 2017, 2024). Ectothermic predators like lizards and spiders tend to select habitats with suitable temperatures to optimize their fitness (Angilletta et al. 1999; Taucare-Ríos et al. 2024). It is generally understood that these organisms reside in areas that align with their preferred temperature (i.e., thermal niche) (Angilletta et al. 1999). In this sense, thermal niche determines where these animals seek refuges that allow them to maximize their reproduction, foraging, and predatory avoidance (Angilletta et al. 1999; Taucare-Ríos et al. 2024). Thermal preferences (*T*_pref_) are obtained in reptiles and spiders using a thermal gradient and represent the temperatures chosen freely in laboratory conditions without biotic interactions (Angilletta et al. 1999; Taucare-Ríos et al. 2024).

Gekkonid lizards and sicariid spiders are ectothermic animals and sympatric competitors of desert faunas in Chile, especially in dune habitats (Pérez and Balta 2011; Taucare-Ríos and Piel 2020). The gecko *Phyllodactylus gerrhopygus* is a nocturnal species that lives on rocky and sandy coasts, which during the day hides under rocks, algae, logs, or remains of anthropic origin and is currently classified as Least Concern (Mella and Reyes 2022). The 6-eyed sand spider *Sicarius thomisoides* is a nocturnal species that lives under rocks in the same sandy environments (Taucare-Ríos et al. 2017). Both are small predators consuming mainly spiders, coleopterans, and other insects that hide under rocks (Pérez and Balta 2011; Taucare-Ríos and Piel 2020). Recent evidence of intra-guild predation between these 2 species has been found (Taucare-Ríos and Piel 2020). The high similarity in the microhabitats they inhabit and their essentially insectivorous diet would imply that these species could compete sympatrically.

The study of predator–prey interactions in desert ecosystems associated with microhabitat selection is very important in the ecology of organisms such as small lizards and arthropods (Vidal et al. 2011; Taucare-Ríos and Piel 2020; Taucare-Ríos et al. 2024). These interactions may determine the distribution and abundance of predators and prey in these arid environments (Vidal et al. 2011; Taucare-Ríos et al. 2024). Despite its importance, this type of study has been little developed in the Neotropical region.

Here, we investigate experimental patterns of thermal and microhabitat overlaps between the gecko (*Phyllodactylus gerrhopygus*) (dominant competitor) and the spider (*Sicarius thomisoides*) (subordinate competitor) in a desert landscape. We seek to answer 2 essential questions: Is there niche overlap between these 2 syntopic and sympatric predators? Will there be differences in preferred temperatures and choice of shelters? If both predators show similar preferences (temperatures and microhabitat use) when they are in alopatry, they should show differential utilization of resources when they are in sympatry.

We collected only 10 females for each species in consideration of the capture permits (Captured permission: SAG, Resolución Exenta N°: 6695/2023). Females are more sedentary and spend more time in shelters (Taucare-Ríos et al. 2017; Mella and Reyes 2022). The individuals were found under stones and garbage in the coastline of Punta Gruesa, Iquique, Tarapacá Region, Atacama Desert. After collection, individuals were placed individually in plastic containers (250 cm^3^) containing a small piece of wet cotton. Humidity (65%), temperature (26.6 °C), and photoperiod (12:12) simulated the original conditions of the sampling locality. The animals were fed ad libitum with small *Tenebrio* larvae before starting experiments. Individuals were measured and weighed.

All individuals used here were maintained in the laboratory for 2 weeks. Individuals were kept at 26.6 ± 1.82 °C, RH: 60.2 ± 8.4 under a 12 h light:12 h dark photoperiod and were housed individually in a plastic terrarium. A thermal gradient from 8 to 40 °C was generated in a glass container measuring 50 cm long × 20 cm wide × 20 cm high. This container had a thermoregulated heater (100 W) on one side and a cold point on the other (cooling gels), creating a thermal gradient between the endpoints. This gradient was checked before and during each experiment. Individuals were deposited randomly and were acclimatized in the gradient for 1 h. Both predators were exposed to the thermal gradient at different times of day: morning (10:00) and evening (19:00). Then, the body temperature (represented by dorsal skin temperature) of each individual was recorded every 5 min with an infrared thermometer (EXTECH Instrument, model IR267; IR accuracy: ± 0.2 °C) for 1 h (12 replicates) (Supplementary Table S1). Here, preferred temperature (*T*_pref_) was defined as the thermal niche obtained from the thermal gradient. After each experiment, the glass container was carefully cleaned with ethanol and then, to avoid any remaining, was washed with distilled water.

A total of 10 plastic boxes (30 cm long × 25 cm wide × 10 cm high) with sand were placed in laboratory conditions exposed to direct sunshine during the day. Firstly, adult individuals of geckos (dominant competitor) and spiders (subordinate competitor) (gecko body length: 46.14 ± 8.28 mm; spider body length: 22.22 ± 1.74 mm) were placed in each box simultaneously (i.e., 1 individual per species) (Figure 1A). In the box, only 1 rock was provided as shelter (Figure 1B). The rocks used were flat, 10 cm long, 5 cm wide, and 2 cm high. The next day, the boxes were examined and the number of individual occurrences of each species under refuge was recorded (Figure 1C). Another experiment was conducted with the same individuals, in which only spiders or geckos were placed individually. The boxes were checked every 24 h for 1 week.

**Figure 1:**
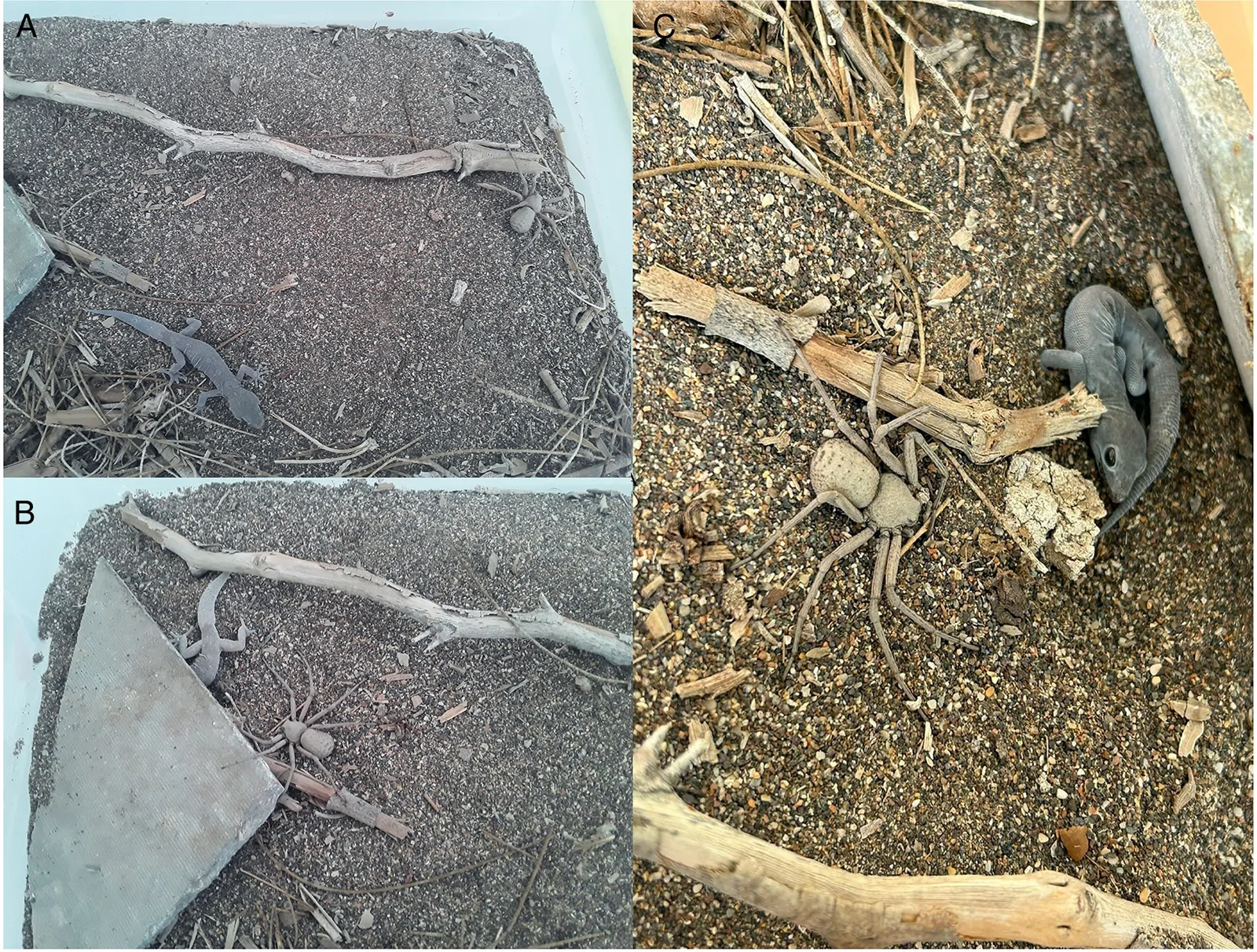
Shelter competition experiment. A) The spider *Sicarius thomisoides* and the gecko *Phyllodactylus gerrhopygus* released simultaneously close to the refuge. B) Both species try to occupy the shelter. C) The next day the rock is lifted to see who is underneath and who is outside.

Before conducting the analyses, we evaluated the assumptions of normality and homoscedasticity of the data (Shapiro–Wilk test and Levene's test, respectively). We used a 2-way repeated-measures ANOVA (Shapiro–Wilk test, *P* > 0.05; Levene's test, *P* > 0.05) to study the preferred body temperature (*T*_pref_) between both species during morning and evening period. Considering that each individual was studied at 2 different hours (repeated-measures design), with *T*_pref_ the response variable and the 2 experimental times (10:00 and 19:00) and the species as the factors. We compare the degree of occupancy of the microhabitat through a Kruskal–Wallis analysis (Shapiro–Wilk test, *P* < 0.05; Levene's test, *P* < 0.05). To check which groups differ, the Dunn test with Bonferroni’s correction was used. We calculated Pianka's index to estimate the degree of microhabitat and thermal niche overlap. This index varies from 0 (no overlap) to 1 (complete overlap) (Pianka 1973). All these analyses were performed using PAST software version 4.0. Data are presented as means ± SE.

In laboratory conditions, the gecko selected a mean temperature of 21.97 ± 5.6 °C and the spider a temperature of 20.69 ± 6.9 °C (2-way repeated-measures ANOVA, *F*_1,18_ = 0.21, *P* = 0.65). Both competitors selected similar *T*_pref_ in different periods of the day (2-way repeated-measures ANOVA, *F*_1,18_ = 1.9, *P* = 0.18) with low temperatures during inactive period (morning) (21.38 ± 1.31 °C) and high temperatures (32.07 ± 1.41 °C) during active period (evening) (2-way repeated-measures ANOVA, *F*_1,18_ = 41.24, *P* < 0.05). We found a high thermal niche overlap (Pianka index = 0.83) and low overlap in microhabitat use (Pianka index = 0.31).

The microhabitat utilization by the gecko in the presence of the spider was 89.9% and increased to 98.9% when the spider was absent but did not differ statistically. The spider occupied the rocks in only 27% of the cases in the presence of the gecko, whereas the spider's occupancy rate increased significantly to 77% in the absence of the gecko (Kruskal–Wallis, *H* = 18.08, *P* < 0.05; Figure 2).

**Figure 2:**
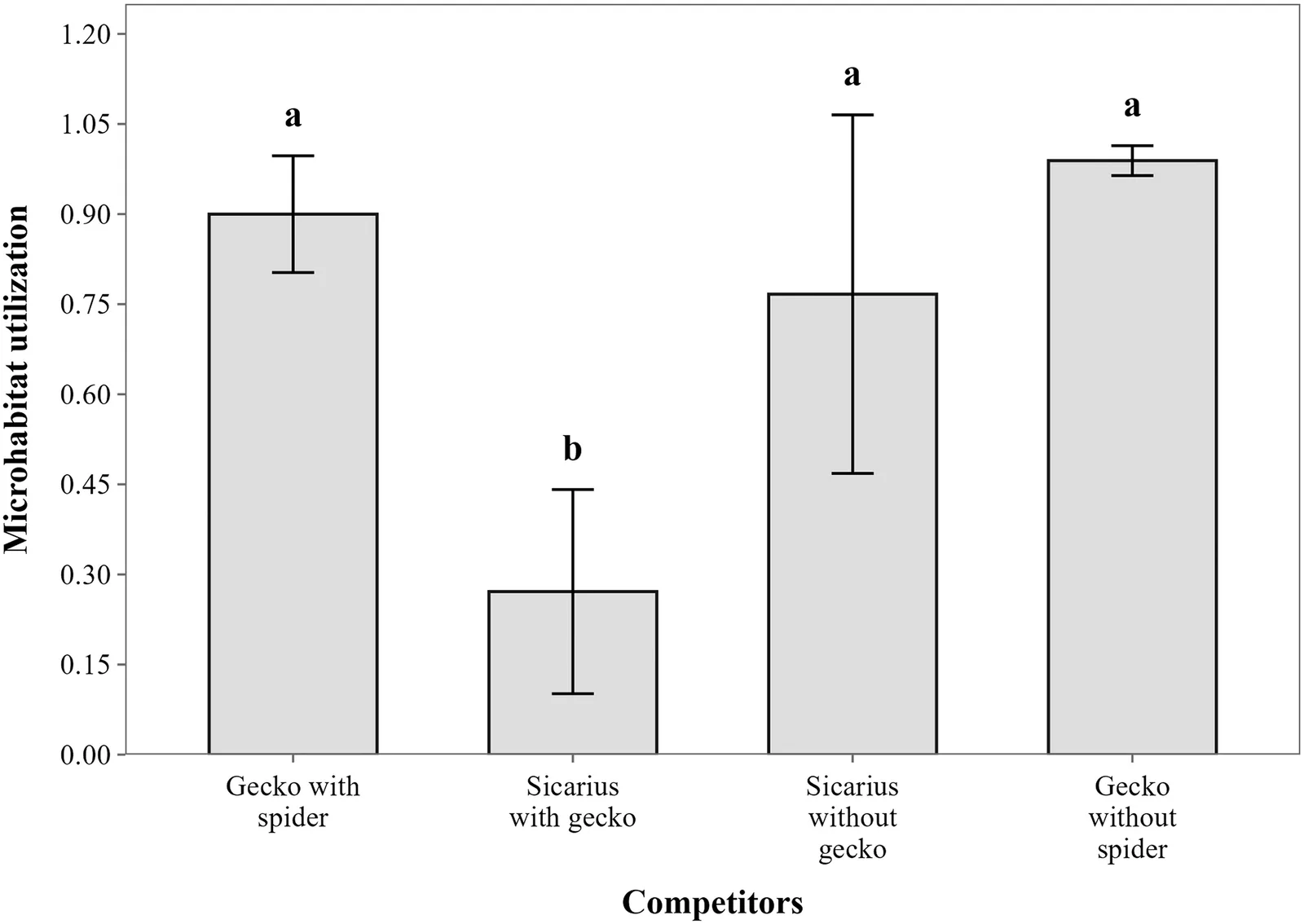
Frequency of microhabitat utilization between the gecko *Phyllodactylus gerrhopygus* and the spider *Sicarius thomisoides.* Different letters assigned to mean values indicate a statistically significant difference (*P <* 0.05), while shared letters indicate no significant difference.

Our results show that both competitors seek similar temperatures and microhabitat in alopatry, but they avoid occupying the same shelters in sympatry. Therefore, species might have adapted strategies to limit direct competition, which might be fundamental for their coexistence (Noble et al. 2011; Pastore et al. 2021). If both species have the same thermal requirements, they are forced to share their refuges. If suitable rocks are a limiting resource, these species must compete to access them.

Both animals increase their body temperature during periods of greater activity. In this sense, nocturnal geckos and spiders tend to select similar temperatures in desert environments, increasing their *T*_pref_ during evening period (Angilletta et al. 1999; Taucare-Ríos et al. 2020). Therefore, increased overlap in thermal niche can be particularly problematic for these competitors because they increase the probability of encounters in their shelters (Pastore et al. 2021; Taucare-Ríos et al. 2024).

Coexistence between predators is characterized by interspecific territoriality, with small species mainly utilizing areas outside the habitat of dominant predators (Harrison et al. 1989). In this sense, the low overlap in the microhabitat may be determined by a niche segregation (Case and Gilpin 1974). Therefore, both predators (gecko and spider) selected similar thermal conditions but avoided the same refuge when are together, suggesting behavioral spatial segregation that may reduce interference.

The results show that spiders significantly avoid sharing shelters in the presence of geckos, suggesting interference competition; however, the avoidance mechanisms are unknown. We suggest the possible presence of chemical signals emitted by both predators, primarily linked to intra-guild predation (Amo et al. 2004; Taucare-Ríos and Piel 2020; Kalmosh et al. 2026). Previous studies in lizards and arachnids have suggested visual and chemical mechanisms for detecting potential predators and competitors (Amo et al. 2004; Kalmosh et al. 2026). Although we did not explore this possibility in this study, probably this mechanism is impacting on the behavior of both species.

An important aspect to consider in this study is that we only chose females for the experiments. Adult females are more sedentary and spend more time inside the shelters (Pérez and Balta 2011; Taucare-Ríos et al. 2017; Mella and Reyes 2022); therefore, it makes sense to use only females. However, one of the main limitations of this study is that we do not know what happens with males and juveniles. In this sense, future research could validate our results including other developmental stages and males, extending our experiments to the field with more complex habitat designs.

In conclusion, we suggest strong competition between these species with the dominance of the gecko, which should lead to competitive exclusion. Both species share similar thermal preferences but show partial microhabitat segregation, possibly to decrease competitive interactions. The gecko appears less affected by interspecific interactions, while spiders are more affected. Future studies should include additional niche dimensions under natural and laboratory conditions to explore the competitive interactions that occur in the field.

## Supplementary Material

zoag007_Supplementary_Data

## Artificial intelligence (AI)-assisted technologies

We did not use artificial intelligence in the manuscript.
